# Bibliographical Notices

**Published:** 1841-12-25

**Authors:** 


### BIBLIOGRAPHICAL NOTICES.

*Introductory Lecture to the Course of Chemistry, delivered in Jefferson Medical College, November* 3, 1841. By FRANKLIN BACAE, M. D.

This is one of the best introductories of the season—a thoroughly well conceived and well written discourse. We should be glad to see it extensively circulated. It might be profitably read, not only by every student of medicine, but by very many professors of chemistry in our medical colleges. Chemistry is a branch much neglected by the student, because often badly taught.

Dr. Bache notices the applications of chemistry to medicine, and first of physiology.

"Although it has been conceded that vital phenomena must be considered as regulated by a distinct set of laws, yet it does not follow that they cannot be elucidated by chemistry. Vital phenomena are the result of the reaction of organic particles ; chemical phenomena, of inorganic ones. Hence physiology and chemistry agree in studying molecular attractions, occurring either within or without the precincts of vitality. As the anatomist separates the grosser parts, and detects differences in structure, so the chemist executes a more minute dissection, by demonstrating the chemical nature of the different animal solids and fluids."

Professor Bache, in entering upon the chair of chemistry, announces his intention,

"To have particular view to the applications of chemistry to medicine. A general view of chemistry will be presented; but those parts of the science which have little or no bearing on medicine will be slightly touched upon; while the medical applications will be fully given."

Thus pointing out the correct mode of teaching his subject, the professor dwells at length upon the importance of its applications to medicine. With much force and point, he illustrates the intimate connection between the two sciences—"a connection which cannot be disregarded by the routine practitioner, much less by the scientific physician." We quote the following just remarks:

"It may be said that much of chemical knowledge is not essentially necessary to the practitioner. It may be alleged that a physician can pursue his profession, without being conversant with chemical physiology and pathology; without knowing the sources and mode of preparation of his chemical remedies; and without being acquainted with the signs of the purity of his medicines. For example, he may prescribe calomel without knowing that it is a metallic preparation, and may, possibly, have studied its physiological and therapeutical effects. I am willing to grant all this: but he may prescribe the same medicine, without knowing that there is such an organ as the stomach! Would this be a valid argument against the indispensable importance of anatomy? No, Gentlemen, we must not listen to such arguments, which, if admitted, would degrade a noble profession below the level of the meanest trade. The students of this college will never condescend to calculate what is the lowest amount of knowledge which may enable them to write a prescription, and drag along in obscurity, with professional acquirements barely sufficient to screen them from merited contempt.

"Chemistry, as a science, has general merits, independently of its application to our profession. Its study enlarges the mind, and presents us with many phenomena, pre-eminently striking and beautiful. Surrounded as we are by the material world, and dependent on it for our comforts, our pleasures, nay, our very existence, we must be sensible how important it is for us to become thoroughly acquainted with the nature of matter. Would it be pardonable in a physician to be unacquainted with the chemical properties of the air we breathe, or of water, which is so essential to the maintenance of life, and which constitutes nine-tenths of our blood, and forms the basis of all the fluids of the body? Independently of the indispensableness of air to life, both animal and vegetable, it is the agent by means of which we speak and hear, and are enabled to perceive odours. Without it, fire could not be sustained, the infant could not draw sustenance from its mother's breast, water could not be raised in a suction-pump, and the familiar operations of leeching and cupping could not be performed."

The tone of this introductory lecture warrants anticipations of a valuable course of lectures from a chemist and teacher of Professor Bache's high reputation—anticipations which we have grounds for saying have thus far been realized.

---

*An Introductory Lecture on the Objects and Nature of Medical Science, delivered in the Hall of the Medical Department of Transylvania University, on the 3d day of November*, 1841. By ELISHA BARTLETT, M. D., Professor of the Theory and Practice of Medicine in Transylvania University.

This is, like all Dr. Bartlett's writings, plain, straight forward, and strictly to the point. We know that such is not the usual fashion of introductory lectures. They are in general intended, not to form part of the course—not even to commence it—but to say some agreeable, and, if possible, striking things, to catch the applause of the class, and make—to use the trivial term—a sensation. We must express our dislike of all this. We do not admire, in a scientific course, any thing which savours of petty expedients, and a highly dressed introductory lecture gives us some distrust of the fitness of the lecturer for a scientific chair. It is true this is not always just. There are men who retain the first rank as teachers of truth, and yet shine as rhetorical writers; and it is pardonable enough for them to indulge their fancy on the only occasion which admits much display of it. Still, there is, in most cases, a connection and resemblance in every thing a man does, which marks, to a certain extent, the character of his mind; and the best augury of an excellent course, is an introductory lecture which announces logical powers of mind in the teacher and a clear happy method of demonstrating the truths of his science, and the immutable laws which govern them, like all other of the phenomena of nature.

